# Mr. Harrup, on Two Diseased Actions at the Same Time

**Published:** 1809-02

**Authors:** Robert Harrup

**Affiliations:** Chobham


### *To the Editors of the Medical and Phyſical Journal.*

GENTLEMEN,

THAT law of the animal œconomy, first promulgated by Mr. J. Hunter, "that no two actions can take place in the same constitution, nor in the same part, at one and the same time," has been eminently illustrated by subsequent observations; particularly by those of Dr. Adams, in his work on Morbid Poisons.

The very few exceptions which have been discovered in the course of a number of years, admitting them to be all correct, only shew how rarely any deviation from this law takes place, so that it may in fact be considered as universal. The inference must therefore be, that one morbid action is capable of suspending another.—"It naturally results, says Mr. Hunter, from this principle, that no two different fevers can exist in the same constitution, nor two local diseases in the same part at the same time."—And a little further on, he, with his usual caution, asks, "Does not the cure of some diseases depend upon the same principle as the suspension or cure of a gonorrhœa by a fever?"* A question which, I believe, may now be answered in the affirmative.

By this knowledge many pathological phenomena, and the modus operandi of various medicines, may readily be accounted for.

Such are the disappearance of almost all other disorders during the prevalence of epidemics and pestilential diseases; and with respect to the operations of different remedies, the use of cold water in fevers, of mercury in certain complaints, and, in many cases, rubefacients, blisters, setons,† &c. may all be referred to the same principle.

Although late writers have in many instances availed themselves

* Vide Treatise on the Blood, Inflammation, &c. p. 3.

† Dr. Underwood, when treating of issues in paralysis of the lower extremities with curvature of the spine, observes, it may indeed be doubted whether the benefit derived from the issue, may not arise rather from the inflammation and stimulus produced on the surface, than from the discharge to which Mr. Pott solely attributes the cure. And in support of this opinion mentions a recent case of an infant, ten months old, where a very sensible relief was afforded as soon as the inflammation took place, and before any suppuration appeared, although the child had been many months a cripple, with loss of health and appetite.—Vide Diseases of Children, Vol. 2, p. 85.

themselves of this doctrine, in order to explain several phenomena, yet I do not find one who has regulated his practice accordingly.—The Editors of the Medical and Physical Journal, in their 20th Vol. p. 464, indeed observe, that "this doctrine of suspending one diseased action by another, has been not only often discovered by accident, but successfully attempted and conducted with design."* I should be glad to know of these gentlemen, if the cases conducted by design have been published, or where they are to be found?

I believe almost every practitioner may be able to recollect numbers of cases that have terminated favourably, which may be easier accounted for on the principle of this doctrine than in any other way; yet it seems singular that in the course of so many years a sufficient number of trials should not have been made, so as to conduct our practice in this respect with some degree of certainty. From this want of experience, no advantage is derived from the knowledge of this law in a practical point of view.

Many years ago, impressed with the belief that the principle of this doctrine, if applied in practice, would be attended with signal advantage, I resolved to attempt it in whatever cases might occur that did not yield to the ordinary remedies. The question I proposed to myself was, may not, in many instances, one disease be suspended or cured by inducing another? In order to induce disease by art, mercury appeared to me to be the most suitable for the purpose in our present state of knowledge, as being that with the effects of which we were best acquainted. For obvious reasons I preferred calomel to the ung. hydrarg. The success of this plan has far exceeded my expectations, and the following cases are only a few of those which have terminated satisfactorily.

1. Mrs. C. upwards of 50 years of age, after removing from a dry comfortable habitation to a damp, low-situated house,

---

* On referring back to the passage, we cannot help feeling surprised that the ingenious author of this paper should require any proof of us. What, as we remarked at the above passage, is the Hunterian theory of the cure of the venereal disease? What is the method of cure in the liver complaint of the East Indies? What is the attempt at curing chronic diseases by inducing gout, so long understood, but at first doubtless discovered by accident?

"So when small humours gather to the gout,
The doctor fancies he has driven them out." Pope.

We mean not by these remarks, to undervalue Dr. Harrup's communications, but to defend our Reviewer's claim to candour. Ed.

house, was attacked with a rheumatic affection, July 1794. Her pains continued to move from one part to another till there were hardly one place of the whole frame which had not suffered; and that which was last affected, invariably continued extremely tender for some time after the pain had ceased. As winter approached her sufferings increased, so that at last she was incapable of moving herself in bed. A great variety of medicines were tried, but with very little success; opium, camphor, and æther only afforded a temporary relief. On the 22d of February, 1795, she began to take a grain of calomel every night and morning. Ten days after her mouth became affected, and a considerable ptyalism ensued, which continued six weeks. She continued free from her complaints during the whole of that time. As the effects of the calomel abated the pains returned, but never with that degree of severity as formerly. Soon after, she went to Bath, and in about two months returned free from complaint.

Several times afterwards she, in damp weather, experienced slight returns of the pains, which readily yielded to one or two doses of æther.

2. Mrs. W. about 75 years of age, after some days indisposition, was seized in the autumn of 1800, with severe pains in the umbilical region; retching and vomiting with obstinate constipation, which was not removed by either cathartics or glysters. Pulse 100 to 108. The third day from the commencement of her complaints, she began taking two grains of calomel with half a grain of opium every fourth hour. Her mouth was affected the following evening, and in a few hours after three large fœtid evacuations from the bowels took place, and her pains entirely subsided. The mouth continued slightly affected about ten days, but she had no further return of the complaint.

3. J. M. Esq. 40 years of age, was attacked for the first time in the latter end of May 1804, with febrile symptoms, pain in the umbilical region, nausea, vomiting, and constipation. The evacuations from the bowels, which were procured with much difficulty by the aid of cathartics and glysters, were extremely fœtid and dark coloured, inclining to green. The disorder continued with more or less violence from May 23, to June 11th. On the 20th he was again attacked in a similar manner, without any evident cause, and which lasted till the 28th. He was again seized on the 21st of July, which did not go off till August 4th. The last and most severe return of the complaint

 commenced commenced October 9th. Towards the beginning of November all his symptoms were aggravated to an alarming degree. On the 3d he began to take two grains of calomel conjoined with a small portion of opium, every five hours, and on the evening of the fifth his mouth was affected, and on the sixth his complaints entirely disappeared. He then only complained of a craving appetite, which he could not satisfy on account of the soreness of his mouth, and a slight tumefaction of the lower part of the face. In eight days the effects of the calomel had nearly subsided, and in two days more he set out for Bath, where, immediately upon his arrival, he experienced a slight return of the complaint, which confined him only a few days. Since that time he has enjoyed a good state of health, excepting once in a few weeks a fit of the sick-head ach, a disorder, to which he has been many years subject.

4. Mr. W. M. 43 years of age, frequently subject to pulmonic affections, with cough, copious expectoration, fever, and night sweats, felt, in the beginning of the year 1806, a particular stiffness and pain in the left knee. Being subject to bad health he considered this as a trifling disorder, only that it impeded his walking in some degree, and therefore used a simple application only to the part. About a month after, when I first examined it, the whole joint was considerably swelled, tense and elastic, and very tender on some parts, particularly on the inner side of the patella, without any discolouration. The tendons in the ham were rigid, and every attempt to move the limb forward was attended with excruciating pain.

Opiates afforded a temporary relief; but no other remedies, whether applied externally or taken, produced any mitigation of the pain. Sometimes, indeed, his sufferings were less acute, and the swelling diminished, but only to return with increased violence. In the early part of the summer the pain began to shoot from the joint to the hip, and continued to increase. He now became emaciated, and lost his appetite; had a cadaverous appearance; a dry troublesome cough; febrile exacerbations and coliquative sweats towards morning. All these formidable symptoms he attributed to want of sleep from excessive pain. It must be confessed, that as no benefit had been derived from any mode of treatment hitherto adopted, I was not sanguine in my expectations that art could afford him relief. Anxious, however, to try as a last resource what could be effected by the exhibition of mercury till the mouth should be affected, he began the 16th of August to take a grain and and a half of calomel conjoined with a grain of opium every night at bed-time, and in the course of the day took two moderate doses of the volatile alkali. After taking the fifth dose of calomel, he complained of slight soreness of the mouth, but took the sixth. The day following a gentle ptyalism came on, which did not increase, and went off in eight or ten days. From the time the affection of the mouth began, his pains abated, and the tumefaction of the joint decreased, so that in the course of a fortnight it was of the natural size, only a small degree of puffiness remained at the upper end of the patella, which soon disappeared. In about a month he was free from complaint, and able to walk without any appearance of lameness.

Last year he had three attacks of the affection of the joint, which invariably yielded to the same mode of treatment. From a particular aversion to mercury, he, after the first time, could not be prevailed upon to take more than five doses of the calomel, and which never failed to affect the mouth in a very slight degree. He has been now several months free from the complaint, and at present takes almost daily exercise to fatigue in the sports of the field.

5. Elizabeth S. 20 years of age, in August 1807 was affected with nausea, loss of appetite, pain of the left side, cardialgia, and other dyspeptic symptoms, during the regular monthly evacuation. In a short time, she was relieved from these complaints by the remedies usually administered in such cases. Soon after the next period all her symptoms returned, and from that time continued gradually to increase, in defiance of every remedy prescribed. Emaciation rapidly succeeded, and the stomach rejected almost every sort of food. No evacuation from the bowels without the aid of cathartics and glysters; the stools of a dark colour, and remarkably fœtid. The left side became tender, and the pain increased; two of the false ribs were considerably raised, and continued so till the time of her death. It is worthy of remark, that during the whole of her illness the pulse seldom varied from the natural standard, till within a short time of her dissolution.

An eminent physician, who visited her occasionally, and prescribed a variety of medicines, which took no effect; at length directed half a drachm of the ung. hydrarg. fort. to be rubbed into the right side every night at bed-time, on the supposition that her disorder was in the liver. She began this treatment November 18th; and on the 22d, having used only two drachms of the ointment, complained of sorenes soreness of her mouth, when the ointment was discontinued. A violent salivation came on, which lasted six weeks. While the effects of the mercury continued, the disease was entirely suspended. The evacuations by the bowels were regular and of a natural colour, and her appetite, which before was entirely gone, became so urgent that she could by no means satisfy the frequent calls of hunger, owing to the soreness of her mouth. As the ptyalism declined, the former symptoms gradually returned, and in a short time after she was much in the same state as before, and died convulsed some months afterwards, having lost the use of her hands and wrists, and in the most extreme state of emaciation.

This case affords a striking example of the power of quicksilver in suspending morbid action, even where internal organic affection has taken place. Is it not highly probable, that this patient might have been saved, had mercury been had recourse to at an earlier period of the disease?

6. Mr. Y. aged 83, in the beginning of the present year, after experiencing some stomach affections, became dropsical. The urine small in quantity and high coloured; the abdomen considerably increased in size, and the lower extremities swelled.

These symptoms yielded to the medicines commonly used in such cases, but soon returned. They were again removed by the same means, and again returned. In May the abdomen and lower extremities were greatly increased. Digitalis and opium, with minute doses of calomel, were taken, but without the least advantage. He now lost all hopes of being benefited by art, and refused to take any medicine. With some difficulty he was prevailed upon to take one grain of calomel conjoined with a very small portion of opium, every night and morning. In ten days his mouth was affected, but in so slight a degree that he could just perceive it. His head swelled considerably, but no increased discharge from the salivary glands took place. The urine was discharged in larger quantity, and in the course of a fortnight he regained his natural size, and experienced no return of the disorder.

7. Mr. G. aged 73, after several weeks confinement by the gout last winter, a disease to which he had been many years subject, discovered symptoms of hydrothorax, from which he was, in some degree, relieved by the squil pill and digitalis. In the spring the abdomen and lower extremities swelled, and respiration was so much affected that he he could not lay down in bed. The former medicines with many others were tried, some of which seemed to give a temporary relief, but no permanent advantage was gained. He then took a grain and a half of calomel twice a day, till his mouth became slightly affected. From that time his complaints began to abate, and soon after disappeared without any return.

8. Mrs. H. about 40 years of age; many years subject to frequent spasmodic affections of the stomach, had for several months perceived a gradual increase of the abdomen, and of the upper and lower extremities, with high coloured urine, much diminished in quantity. As these complaints gave her no farther uneasiness, than an unwieldiness in motion and hurried respiration when walking, she delayed applying for assistance, till they suddenly increased last spring.

Although her appearance indicated more of obesity than actual disease, her limbs felt remarkably hard, and retained the impression of the finger for some time. She took a variety of medicines without any advantage, as her complaints gradually became worse. In the beginning of June she took two grains of calomel daily, till the mouth was gently affected. From that time her symptoms began to abate, and in about four weeks she had diminished to her natural size, and is at present in a good state of health.

9. Mrs. S. 35 years of age, of a delicate constitution, and subject to frequent miscarriages, was greatly alarmed by supposing she had stepped upon a viper. The menses, which were present at the time, suddenly increased to a high degree, and strong hysterical symptoms came on. By quiet and some anodyne medicines, she soon recovered, and the menstrual discharge disappeared gradually. Two or three days afterwards, she was suddenly attacked by the following symptoms. First a violent pain of the loins; a death coldness of the hands and feet; the countenance much changed, and highly expressive of anguish. Voice querulous with constant moaning; jactitation; pulse 96 to 102, and feeble; micturition difficult; the urine voided in small quantities and high coloured, with constipated bowels. In a few hours the pain extended to the abdomen, which was succeeded by violent retchings, and a discharge of a bitter and acid fluid. The whole of the abdomen, from the scrobiculus cordis to the pubis, and indeed wherever the pain extended, were exquisitely tender. In this miserable state she continued eight days, when the belly became somewhat swelled, and her complaints abated.

Opiates

Opiates afforded little relief; antispasmodics, cathartics, blisters, and fomentations were attended with no benefit, and glysters gave but a momentary ease. Before she had quite recovered from the effects of this severe illness, the catamenia appeared at the regular time, and continued a few days as usual. Two days after they ceased, she was seized exactly in the same way as in the preceding month, only the symptoms were, if possible, more severe. In addition to the former remedies, the warm bath was now had recourse to, which did not fail to give ease during the immersion, and for a short time after. In eight days the complaint began to diminish, and at last disappeared in the same manner as before.

As the disorder appeared to be periodical, and considering the debilitated state to which she was reduced, it seemed highly probable that the next attack would prove fatal, if prompt means were not had recourse to, at the commencement, to stop its progress. The remedies hitherto tried, had been of very inconsiderable service. I therefore resolved to affect the mouth by calomel, at the beginning of the next attack. This will undoubtedly be thought a bold practice; but I considered myself justified in the attempt, from what I had observed in other cases; and concluded that no other probable means were left to save the patient.

The same train of symptoms, with equal severity, again recurred on the afternoon of the 20th of last August, at the time I expected. In the evening she began to take a pill, every fourth hour, composed of one grain of calomel and a quarter of a grain of opium. Forty-eight hours after, she complained of tenderness and bleeding of the gums, but experienced no alleviation of the pains and other symptoms. The pills were now omitted. Next morning I was much gratified to find her free from complaint, excepting much soreness of the abdomen, and the *disease* occasioned by the calomel. Ptyalism had taken place, and continued three weeks. The only complaint she had during this time was the affection of her mouth, which prevented her gratifying a craving appetite for food.

She has been seized twice since with the same symptoms, at the usual periods, but in so slight a degree that she was not confined more than two or three days each time, and which readily yielded to a few doses of camphor in substance, and jalap, neither of which procured any benefit in the former attacks. She has menstruated once since, but without without any unusual symptoms, and is at present in a good state of health.*

Although I have employed calomel in this manner, with much success in various diseases, and in a great number of cases besides these just related; yet I wish not to be considered as an advocate for salivation, or even a slight affection of the mouth on every trivial occasion, or where there may be a prospect of benefit by other means. In my own practice, I have never had recourse to it as a sialagogue, till other remedies had proved ineffectual; and then, in many cases, the affection of the mouth was so gentle, but yet sufficient, that the patient was ignorant of the cause. Undoubtedly, many obstinate and severe cases will occur, where the action of this mineral preparation must be pushed much farther, in order to succeed, and kept up for some time, particularly in chronic diseases.

We can never be certain of the action of mercury, on the system in general, till it has affected the mouth, and in this respect it is the best index to our practice.

As a particular objection to quicksilver, it has been frequently

* Mr. Abernethy, whose accuracy of observation is unquestionable, in his Surgical Observations, Part 2d. p. 69, when treating of disorders of the digestive organs, remarks, that he has observed in some instances, where small doses of mercury have unexpectedly affected the mouth, that considerable benefit seemed to arise from this circumstance. In the same volume, p. 196, a case is related, which is so much to the purpose, that I take the liberty of giving it in his own words. "A servant of mine, says this gentleman, told me, that his wife was dying of a consumption, which had been rapidly increasing for six months, and had baffled all attempts to relieve it. Thinking that I could procure her some medical assistance from the hospital, I went to see her. The case, however, seemed passed hope. She was extremely emaciated, her pulse beat 140 in a minute; her face was flushed; she had a most distressing cough; and spit up more than a pint of mucus, mixed with pus and streaked with blood, in twenty-four hours. The circumstance, however, which most disturbed her, was a continual purging of black and offensive matter. She told me that the disorder of her bowels was the first disorder; that it had preceded the pulmonary affection, and indeed, that it was an habitual complaint. I thought it unnecessary to trouble my medical friends in so hopeless a case, and ordered some pills, containing one grain of opium, to be taken in such quantity as was necessary to stop the purging. As she informed me that the disorder began in the bowels, I added to each pill half a grain of calomel. By these means the purging was so much checked, that she did not find it necessary to take more than two pills in twenty four-hours; and when she had taken twelve, the mercury very unexpectedly affected the mouth. From that period, the stools became of a natural colour and consistence, the cough and expectoration ceased; and she was soon sufficiently recovered to go into the country; from whence she returned apparently in good health."

quently urged that its use is highly injurious to the constitution.

Although I have never met with an instance of this kind, where the complaints could be fairly attributed to the remedy, yet I have no doubt but such cases do sometimes occur. I am persuaded, however, that the generality of instances adduced in support of this opinion, will be found to arise more from some fault of the constitution, than from the effects of the medicine. In a number of venereal cases, I have known the use of mercury carried to a most extravagant length; and the unfortunate patients suffer greatly for the time; but I never found that one of them afterwards experienced any of those melancholy symptoms, which are supposed to be induced by such treatment. The largest quantity I ever heard of, used by any person, was by a syphilitic patient, who at last put himself under my care about four years since. The quantity of the stronger ointment he used in the space of two months was immense, besides calomel internally to a great amount. He was reduced to the lowest state of debility and emaciation and had eleven large spreading foul ulcers on his body. By the help of the nitric acid and other medicines, he recovered in three months. Since then he has continued free from any complaint, and is at present one of the most robust and healthy men with whom I am acquainted.

That calomel is successfully employed in various diseases is well known. Such as in the bilious remittent fevers of hot climates; in the yellow fever by Drs. Rush and Chisholm; in hepatitis, dysentery, &c. and continued till the mouth becomes affected. The different preparations of quicksilver, whether used externally or internally, are also found to be powerfully antispasmodic, and as such are employed in all affections of this kind. These, and the many other virtues, which the mineral is known to possess, can be satisfactorily accounted for in no other way, than by its power of inducing an action different from that which already exists. If this doctrine be admitted, other remedies may perhaps be found, that will produce the same salutary effects.

It has been observed at the beginning of this paper, that cold affusion, under certain circumstances, in fever, and some other remedies, act in this manner. Without attempting to swell the catalogue, in order to avoid cavilling, I shall only notice two others, viz. Emetics and blood-letting. The influence of emetics on the system is so extensive and powerful, that they have been employed for for ages in various diseases. To enumerate, in this place, all the virtues ascribed to them, would be unnecessary, as they are recorded in every work on the materia medica. Few practitioners, if any, consider them to be of no other service than to evacuate the contents of the stomach; but I believe many imagine that their most salutary effects arise from their determining to the skin. To preserve the external surface in a proper state of relaxation, is certainly desirable in all cases; but we find emetics producing effects far beyond that of any other sudorific or diaphoretic medicine. It appears evident to me that their efficacy arises chiefly from their power of suspending morbid action, by the sickness and consequent debility which they induce; hence their power in removing fever when taken at the commencement; in suspending the paroxysm of an intermittent; and, when administered in nauseating doses, in checking pulmonary and uterine hæmorrhagies, &c. &c.

The utility of abstracting blood from the system in one class of diseases is so manifest, that there can be only one opinion on the subject; but there are also many complaints in which the use of the lancet is so doubtful, that the greatest caution is necessary. In all such cases we shall often find that blood-letting proves beneficial to some patients, and to others either injurious or of no effect.

Perhaps, by reasoning on the modus operandi of this remedy, we may be somewhat assisted in determining what constitutions, under certain states of disease, may be benefited by it.

Dr. A. Monro gives two cases in the Edinburgh Medical Essays,* in which the patients were cured by losing a large quantity of blood by accident. The one, of a child, eight months old, who was seized with epileptic fits, and after having several, had five ounces of blood taken from the jugular vein, and soon after being put into the cradle lost a considerable larger quantity; she continued weak and pale several days, but never had any return of the epilepsy. The other, that of a woman in the decline of life and of a broken constitution, who had narrowly escaped out of a rheumatic fever after several weeks confinement, and two years afterwards was seized with the same symptoms, when fourteen ounces of blood were taken from a vein of the arm, which gave her little or no relief. In the night time the blood burst out again at the orifice, and besides wetting all the bed-clothes, was lying in clots at her

* Vide Vol. VI. p. 25.

side, before it was taken notice of. A clean bandage being then applied she fell asleep, and awaked next morning free of all complaints.

In the last Number of the Medical Journal, a publication of cases of diabetes, consumption, &c. by Dr. Watt, is reviewed. By the extracts there given, it appears that a number of cases of diseases of debility, at least which are generally considered as such, were either cured or greatly relieved, chiefly by abstracting blood in considerably large quantities; and Dr. Kinglake, in the preceding number, observes, that "a well regulated course of bleeding, in the early stage of phthisis pulmonalis, appears to him to afford more unequivocal benefit, than any other mode of assistance." In all such cases, I apprehend, that the effects of abstracting blood may be explained on the principle of suspending morbid action. Thus, in diseases of debility, when a certain quantity of blood is taken away, the re-action of the system will, for a time, suspend the morbid action, but if the re-action be too feeble for this purpose, the patient must infallibly be injured by the operation.

A fact, noticed by Dr. Pemberton, in his Practical Treatise on Various Diseases of the Abdominal Viscera, will tend still farther to illustrate the doctrine here contended for. After observing that physicians have been struck at all times with the effect produced, by taking the blood from a large orifice in inflammatory diseases: It is true, continues he, that from a *small* orifice the same quantity of blood may be taken as from a *large* one, but the *time* of its flowing is so long, that the topical inflammation, which demands for its relief a *sudden effect upon the system,* is not in the least influenced by it, though the general strength is much weakened, &c. &c.*

In short, were I to hazard a general proposition on the subject, it would be, *that in every case where a different action is induced it will either suspend, or entirely remove the morbid action then existing.* Of this many instances may be found recorded by different authors, in which cures have been effected by very unexpected means.

Whatever may be the opinions entertained of this doctrine, of which I have thus attempted a slight outline, it were much to be wished that practitioners would bear in mind the principle I set out with, in their practice, and give to the public, through the medium of the Periodical Journals, the result of their observations.

